# Arthralgien und Uhrglasnägel führen zur Diagnose eines Bronchialkarzinoms

**DOI:** 10.1007/s00393-025-01752-7

**Published:** 2025-11-26

**Authors:** Franca Deicher, Laura-Marie Lahu, Wolfgang Merkt

**Affiliations:** 1 Klinik für Rheumatologie, Universitätsklinikum Düsseldorf, Medizinische Fakultät, Heinrich-Heine Universität, Moorenstr. 5, 40225 Düsseldorf, Deutschland; 2 Hiller Forschungszentrum, Universitätsklinikum Düsseldorf, Medizinische Fakultät, Heinrich-Heine Universität, Düsseldorf, Deutschland; 3 Klinik für Hämatologie, Onkologie und Rheumatologie, Innere Medizin V, Universitätsklinikum Heidelberg, Heidelberg, Deutschland

**Abb. 1:**
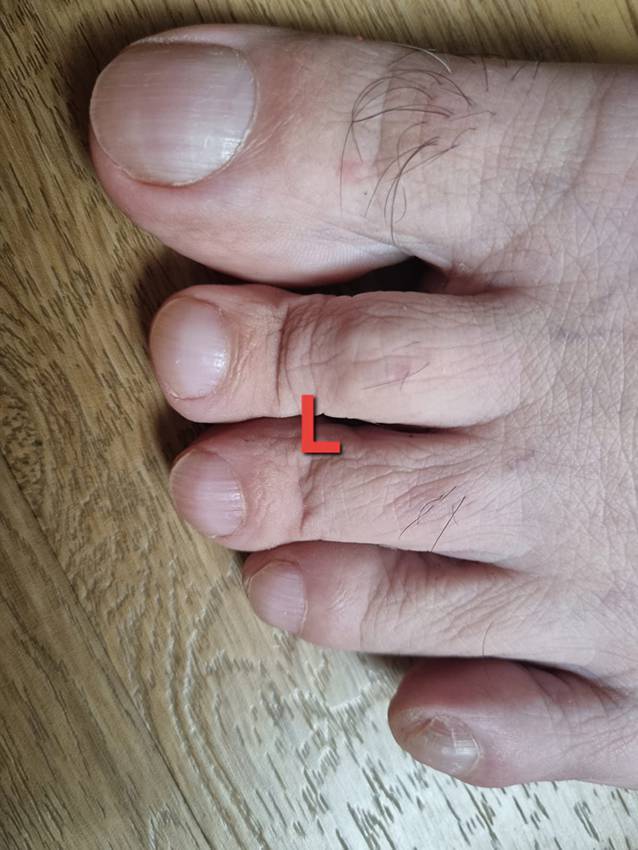
Foto des linken Fußes mit Uhrglasnägeln und Trommelschlägelzehen

Ein 72-jähriger Mann stellte sich in unserer rheumatologischen Abteilung mit ausgeprägten Arthralgien vor, die mehrere Gelenke betrafen (Knie > Schulter > Sprunggelenk). Laborchemisch zeigte sich ein erhöhter CRP-Wert von 8 mg/dl, eine leichte normozytäre Anämie und eine Thrombozytose, während serologische rheumatologische Untersuchungen keine signifikanten Befunde ergaben. In der Arthrosonographie kamen leichte Gelenkergüsse ohne weitere direkte oder indirekte Anzeichen einer Arthritis zur Darstellung. Stattdessen ergab die klinische Untersuchung auffällige Trommelschlägelfinger und Uhrglasnägel an Fingern und Zehen (Abb. 1 und 2), die der Patient erstmals vor 6 Monaten bemerkt hatte. In Verbindung mit einer Raucheranamnese von 40 packyears und auf Nachfrage berichtetem Husten im letzten Jahr vermuteten wir eine mögliche paraneoplastische hypertrophe Osteoarthropathie (Pierre-Marie-Bamberger-Syndrom). Die Röntgenaufnahme bestätigte eine hypertrophe Osteoarthropathie an typischen Stellen, z. B. am rechten Femur (Abb. 3 und 4). Schließlich zeigte die CT der Lunge eine große Tumorläsion (5,4 cm) im rechten Oberlappen und eine ausgeprägte Hiluslymphadenopathie (Abb. 5 und 6). Die Diagnose eines nichtkleinzelligen Lungenkarzinoms (Plattenepithelkarzinom, Pancoast-Tumor) wurde durch eine Biopsie bestätigt.

**Abb. 2:**
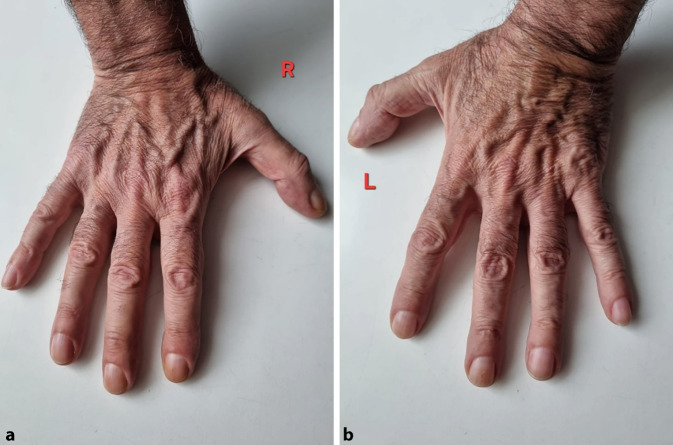
Fotos der Hände (**a** rechte Hand, **b** linke Hand) mit Uhrglasnägeln und Trommelschlägelfingern

**Abb. 3:**
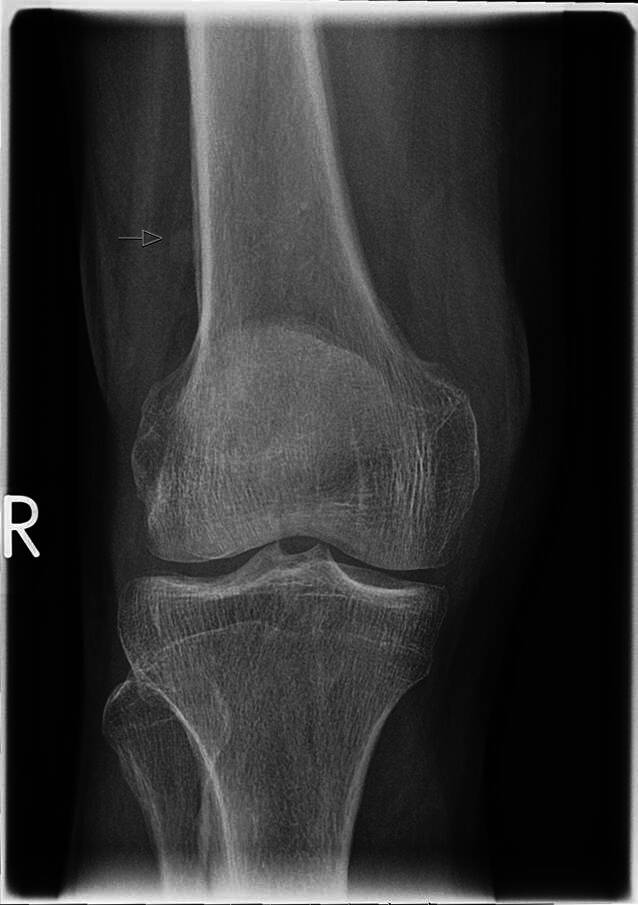
Röntgenbild des rechten Knies mit typischen periostalen Anbauten des Femurs

**Abb. 4:**
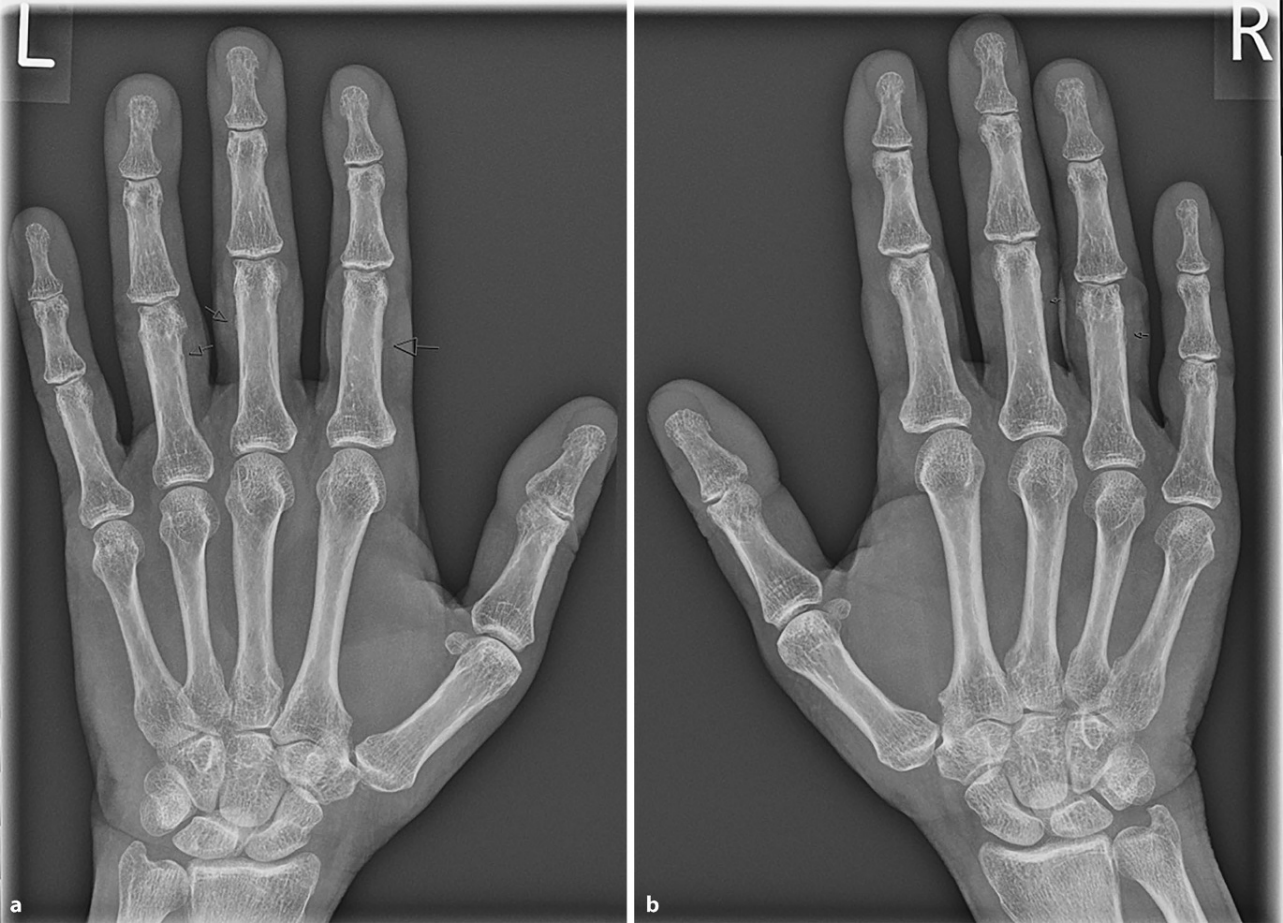
Röntgenbilder der Hände (**a** linke Hand, **b** rechte Hand) mit typischen periostalen Anbauten

**Abb. 5:**
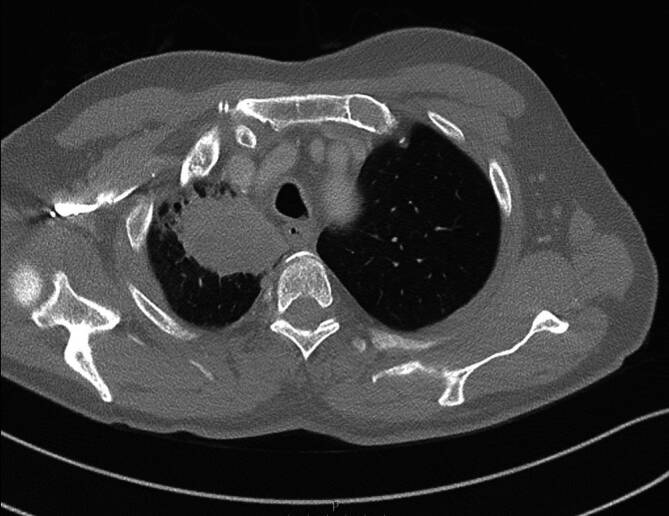
Transversale CT Thorax mit apikaler pulmonaler Raumforderung

**Abb. 6:**
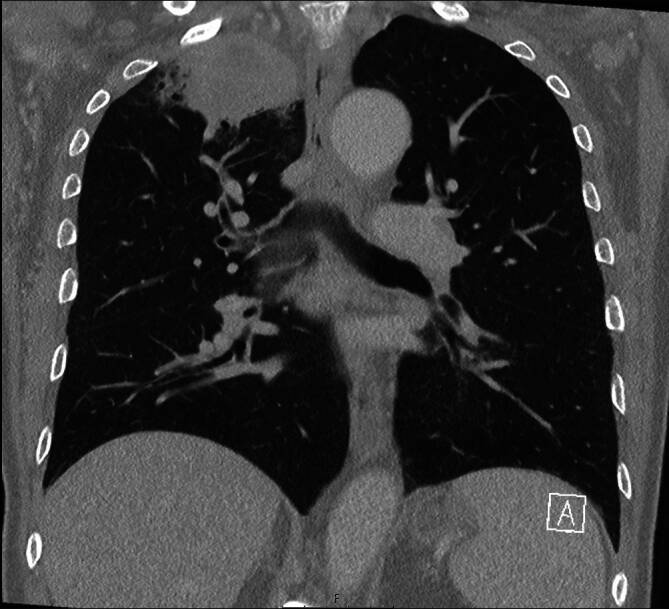
Frontale CT Thorax mit apikaler pulmonaler Raumforderung

Arthralgie in Kombination mit Trommelschlägelfingern und -zehen als klinische Anzeichen einer hypertrophen Osteoarthropathie bei Rauchern können die ersten Symptome eines Bronchialkarzinoms sein und sollten zu weiteren Untersuchungen der Lunge führen.

